# Gene expression of *CCL8* and *CXCL10* in peripheral blood leukocytes during early pregnancy in cows

**DOI:** 10.1186/s40104-018-0263-z

**Published:** 2018-06-20

**Authors:** Ryosuke Sakumoto, Kosuke Iga, Ken-Go Hayashi, Shiori Fujii, Hiroko Kanahara, Misa Hosoe, Tadashi Furusawa

**Affiliations:** 1 Division of Animal Breeding and Reproduction Research, Institute of Livestock and Grassland Science, National Agriculture and Food Research Organization (NARO), Ibaraki, 305-0901 Japan; 2 Division of Livestock and Forage Research, Tohoku Agricultural Research Center, NARO, Morioka, 020-0198 Japan; 3 Division of Animal Sciences, Institute of Agrobiological Sciences, NARO, Ibaraki, 305-8602 Japan

**Keywords:** CCL8, Cow, CXCL10, Leukocytes, Pregnancy

## Abstract

**Background:**

The aim of the present study was to evaluate *CCL8* and *CXCL10* expression and its regulatory mechanism in peripheral blood leukocytes (PBLs) at the time of maternal recognition in cows. Blood samples were collected on 14, 15, 16, 17 and 18 d after artificial insemination (AI). Based on the day of return of estrus, cows were divided into three groups, pregnant (*n* = 5), early embryonic mortality (EEM; *n* = 5) and late embryonic mortality (LEM; *n* = 5). The gene expression levels in PBLs were assessed with quantitative real-time reverse transcription PCR.

**Results:**

The expression of *CCL8* and *CXCL10* mRNA in PBLs gradually increased from 14 to 18 d of pregnant cows and significant differences were observed on 18 d (*P* < 0.05), whereas no significant changes were observed both in EEM and LEM cows. Interferon-stimulated protein 15 kDa (*ISG15*), myxovirus-resistance gene (*MX*) 1 and *MX2* mRNA expression in PBLs increased from 14 to 18 d which was significant on 18 d of pregnant cows as well as in LEM cows (*P* < 0.05), but no changes were observed in EEM cows. To determine whether the expression of *CCL8* and *CXCL10* in PBLs was regulated by pregnancy-related substances or not, expression level was assessed after exposure to interferon-τ (IFNT) and CCL16. Monocytes, granulocytes and lymphocytes were obtained using density-gradient centrifugation and flow cytometry. The addition of IFNT (100 ng/mL) and CCL16 (100 ng/mL) to cultured PBLs increased the expression of *CCL8* and *CXCL10* mRNA (*P* < 0.05). The expression of *ISG15*, *MX1* and *MX2* mRNA in PBLs was also stimulated by IFNT and CCL16 (*P* < 0.05).

**Conclusions:**

The expression of *CCL8* and *CXCL10* genes increased in PBLs during early pregnancy. Since IFNT stimulated *CCL8* and *CXCL10* expression in cultured PBLs, the increase of CCL8 and CXCL10 might be pregnancy-dependent events. The expression of both *CCL8* and *CXCL10* in PBLs was stimulated by CCL16 as well as IFNT, suggesting a chemokine interaction that at least includes CCL8, CXCL10 and CCL16, and may play a role in regulating maternal recognition in cows.

## Background

Diagnosis of early pregnancy is important to improve reproductive efficiency and bring economic benefits to livestock production [1]. The ability to detect pregnancy early would help to shorten interbreeding intervals, since nonpregnant cows can be given an opportunity to synchronize their estrous cycle and be artificially inseminated prior to the next ovulation. Over the past few decades, various direct or indirect methods to diagnose pregnancy have emerged such as rectal palpation [2], ultrasonography [3], serum progesterone concentration [4], early pregnancy factor [5], and pregnancy-specific protein B (PSPB: known as pregnancy-associated glycoprotein-1) [6, 7]. Although these methods to diagnose pregnancy have individual merits, they can only be adopted at the earliest 4 wk after artificial insemination (AI).

Recent studies have shown that interferon-τ (IFNT), which is a known key maternal recognition factor, stimulates the expression of interferon-stimulated genes (ISGs), such as interferon-stimulated protein 15 kDa (ISG15), myxovirus-resistance (MX) proteins 1 and 2, and 2′-5′-oligoadenylate synthetase 1 (OAS1) in peripheral blood leukocytes (PBLs) in cows [8–14]. These findings provide a possibility to diagnose pregnancy within 3 wks after AI, but have shown variable effectiveness. For example, *ISG15* mRNA levels in PBLs on 18 d after AI revealed poor accuracy as an early pregnant marker in dairy cows, although accuracy increased from 17 to 25 d [8]. *MX2* mRNA levels in PBLs from 0 to 18 d after AI did not change considerably in dairy cows [9], whereas *OAS1* mRNA levels were successfully used to assess pregnancy on 18 d in heifers but not in cows [10]. Furthermore, a combination of gene expression of *ISGs* in PBLs and color Doppler ultrasonography of the corpus luteum on 20 d after AI in beef cattle was used as a feasible method to diagnose pregnancy with high accuracy [14].

Our recent study demonstrated that the expression of *CCL8* (also known as MCP-2) and *CXCL10* (also known as IP-10) mRNA was higher in the bovine endometrium on 15 and 18 d of pregnancy than in the non-pregnant stage [15]. Furthermore, the expression of both *CCL8* and *CXCL10* increased after stimulation with IFNT using an in vitro endometrial culture system [15]. We subsequently hypothesized that the expression of *CCL8* and *CXCL10* would increase in PBLs during early stages of pregnancy, and that it was possible to use both chemokines as biomarkers for early pregnancy diagnosis in cows. In the present study, we therefore examined changes in the expression of *CCL8* and *CXCL10* in PBLs obtained from cows on 14 to 18 d after AI in three conditions: pregnancy, early embryonic mortality (EEM) or late embryonic mortality (LEM). The effects of IFNT and CCL16, which stimulated *ISGs* mRNA expression in the bovine endometrium [15], on the expression of *CCL8 *and *CXCL10* by monocytes, granulocytes and lymphocytes fractionated by flow cytometry were also studied.

## Methods

### Collection of bovine peripheral blood

Fifteen normal cyclic Japanese Black cows (age: 3–13 yr, parity: 1–9) were used in this study. To detect estrus, cows were observed daily for standing behavior. AI was performed on the day of estrus using frozen semen from Japanese black bulls. The day of AI was designated as 0 d, and blood was collected from the jugular vein in heparin sodium-containing vacutainers on 14, 15, 16, 17 and 18 d after AI. Half of the blood samples were centrifuged at 1,200×*g* for 30 min, and plasma was separated to evaluate protein concentration. The remaining of blood and plasma samples were stored at − 80 °C. The data were collected separately for the pregnant (confirmed parturition; *n* = 5), EEM (return of estrus during 19–21 d; *n* = 5) and LEM (return of estrus during 25–35 d; *n* = 5) cows, according to the criteria of a previous study [16].

### Real-time PCR

Total RNA was isolated from whole blood using a commercial RNA extraction kit (NucleoSpin RNA Blood, #740200, Macherey-Nagel GmBH&Co. KG, Büren, Germany) in accordance with the manufacturer’s instructions. The NucleoSpin RNA blood kits are recommended for the isolation of RNA from fresh and frozen whole blood. All RNA samples were quantified by spectrophotometry (#ND-1000, Nanodrop Technology Inc., Wilmington, DE, USA) and the purification of RNA with A260/A280 ratio was between 2.0 and 2.2. Complementary DNA was synthesized from 500 ng of total RNA using a QuantiTect Reverse Transcription kit (#205314, Qiagen, Hilden, Germany). Gene expression was measured by real-time PCR using an Mx3000P Real Time PCR analyzing system (Agilent Technologies, Santa Clara, CA, USA) and a QuantiFast SYBR Green PCR kit (#204054, Qiagen) as previously described [17]. The primers encoding the bovine sequences were chosen using an online software package (http://primer3.ut.ee/) and synthesized as listed in Table 1. Primer length (18–21 bp) and GC content of each primer (50% to 60%) were selected to avoid primer dimer formation. PCR was performed under the following conditions: (first step) 95 °C for 5 min; 45 cycles of 95 °C for 15 s, 60 °C for 30 s and (second step) 95 °C for 60 s; then 60 °C for 30 s. The reaction was then held at 25 °C. After each PCR cycle, melting curves were obtained to ensure single product amplification. Standard curves for each gene were generated by serially diluting plasmids containing cDNA of each individual gene to quantify mRNA concentration. The obtained data were normalized on the basis of *GAPDH* mRNA content. To exclude any contaminating genomic DNA, all experiments included controls that lacked the reverse transcription enzyme. As a negative control, water was used instead of RNA for PCR to exclude any contamination from buffers and tubes.

**Table 1:** Primers used in real-time PCR

Genes		Sequence (5′→3′)	GenBank accession number	Size, bp
*CCL8*	Forward	AACATGAAGGTCTCCGCTGG	NM_174007	108
	Reverse	GCAGCAGGTGATTGGGGTAG		
*CXCL10*	Forward	CTCGAACACGGAAAGAGGCA	NM_001046551	117
	Reverse	TCCACGGACAATTAGGGCTT		
*ISG15*	Forward	GCAGACCAGTTCTGGCTGTCT	NM_174366	58
	Reverse	CCAGCGGGTGCTCATCAT		
*MX1*	Forward	GAGGTGGACCCCCAAGGA	NM_173940	58
	Reverse	CCACCAGATCGGGCTTTGT		
*MX2*	Forward	GGGCAGCGGAATCATCAC	NM_173941	55
	Reverse	CTCCCGCTTTGTCAGTTTCAG		
*GAPDH*	Forward	ACCCAGAAGACTGTGGATGG	U85062	158
	Reverse	CAACAGACACGTTGGGAGTG		

### Flowcytometric collection of monocytes, granulocytes and lymphocytes

Flowcytometric separation of PBLs was carried out as previously described [13] with a slight modification. Blood samples were collected by the jugular vein from five Japanese Black cows on 10–12 d of the estrous cycle. After blood was collected, 8 mL of whole blood was carefully layered onto 4 mL of Histopaque-1119 (1.119 g/mL density, Sigma-Aldrich Co., LLC, St. Louis, MO, USA) and centrifuged at 780×*g* for 30 min at room temperature. The serum fraction was removed. Two milliliters of the upper part of the whole blood cell pellet fraction, which included red cells, was centrifuged at 1,200×*g* for 5 min at room temperature. After removing the supernatant, the blood cell fraction was suspended in pre-warmed (37 °C) lysing buffer (155 mmol/L NH_4_Cl, 10 mmol/L KHCO_3_, and 1 mmol/L EDTA), and immediately diluted with sorting buffer composed of Hank’s balanced salt solution containing 2% fetal bovine serum and 10 mmol/L 4-(2-hydroxyethyl)-1-piperazineethanesulfonic acid. After washing with sorting buffer, cells were re-suspended in cold sorting buffer containing 2 μg/mL propidium iodide (PI) and sorted into three populations (monocytes, granulocytes and lymphocytes) using a MoFlo Astrios cell sorter (Beckman Coulter, Carlsbad, CA, USA). Cell debris and platelet populations were removed using several intrinsic fluorescent parameters and cells were then sorted using the 488–513/59 parameter versus side scatter (SSC) or the 355–448/59 parameter. Each cell population was confirmed by cell lineage-specific antibodies: anti-granulocyte antibody (#MM20A, VMRD Inc., Pullman, WA, USA), anti-bovine monocyte antibody (#BAQ151, VMRD), or anti-CD3 antibody (#MM1A, VMRD).

### Culture of PBLs

The monocytes, granulocytes and lymphocytes were placed separately in culture medium (DMEM/Ham’s F-12; 1:1 (*v*/*v*); #D8900, Sigma-Aldrich) supplemented with 10% (*v*/*v*) calf serum (#C6278, Sigma-Aldrich), 20 IU/mL penicillin, 20 μg/mL streptomycin, and 0.05 μg/mL amphotericin B (#516104, EMD Millipore Corp. Billerica, MA, USA) and cultured at 37.5 °C in a humidified atmosphere of 5% CO_2_ in air (2.5 × 10^4^ cells/200 μL/well in a 96-well culture plate, Nunc-Thermo Fisher Scientific). Cultured leukocytes were further incubated in this medium with recombinant proteins as follows: bovine IFNT (100 ng/mL: 1.1 × 10^5^ units/mg, generated from HEK293 cells as described previously [18]) or recombinant human CCL16 (100 ng/mL: #TP723266, OriGene Technologies, Inc., Rockville, MD, USA). After incubation for 18 h, the leukocytes and supernatant were collected separately and stored at − 80 °C until use. Total RNA from PBLs was extracted using an RNeasy Micro Kit (#74004, QIAGEN) in accordance with the manufacturer’s protocols, and used for the subsequent gene expression analysis.

### Statistical analyses

The expression ratio of each gene to *GAPDH* mRNA was calculated to adjust for variations in the PCR reaction. The experimental data for real-time PCR are presented as the mean ± SEM. The difference of mRNA expression in the PBLs between 14 d and 15, 16, 17, 18 d in each group (pregnant, EEM and LEM) was analyzed using one-way ANOVA for repeated measures with Dunnett’s Multiple Comparison post hoc test with the KaleidaGraph 3.6 (Synergy Software, Reading, PA, USA) software package. In PBL culture experiments, the experimental data for real-time PCR were shown as percentage of the control value (*n* = 5). The differences between mRNA expression in the cultured PBLs between the control group and the treated group were analyzed using one-way ANOVA with Tukey-Kramer multiple comparison test. A *P*-value < 0.05 was considered statistically significant.

## Results

**Fig. 1:**
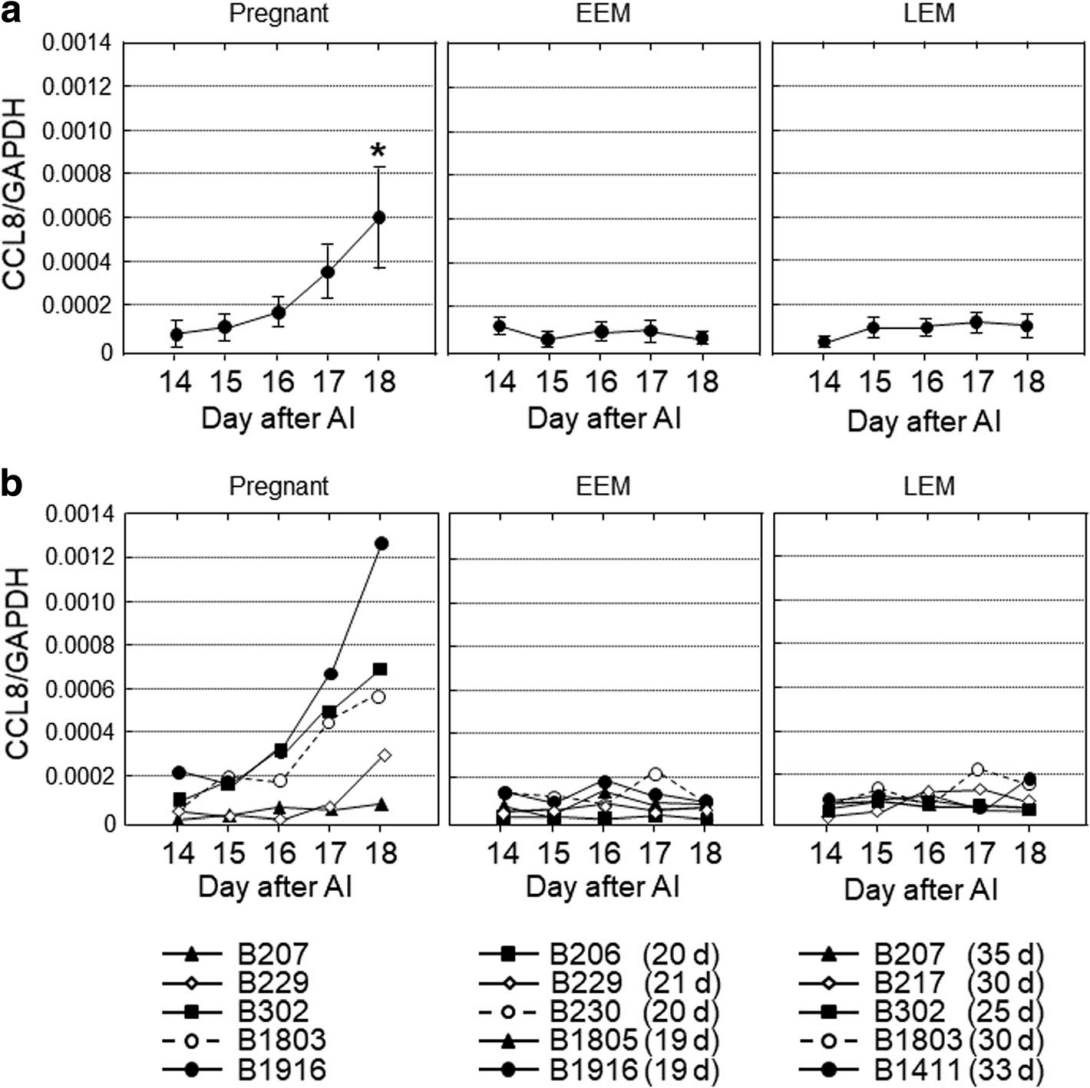
The gene expression levels of *CCL8* in peripheral blood leukocytes obtained from cows on 14, 15, 16, 17 and 18 d after artificial insemination (AI). Based on the day of return of estrus, the cows were divided into three groups, pregnant (*n* = 5), early embryonic mortality (EEM; *n* = 5) and late embryonic mortality (LEM; *n* = 5). **a** Data are means ± SEM of five cows and are expressed as relative ratios of the mRNAs to *GAPDH*. Values significantly different from the value on 14 d after AI are shown with an asterisk (*P < 0.05). **b** Each line represents the data for individual cows, and the figure in parentheses indicates the day of the return of estrus in EEM and LEM cows

**Fig. 2:**
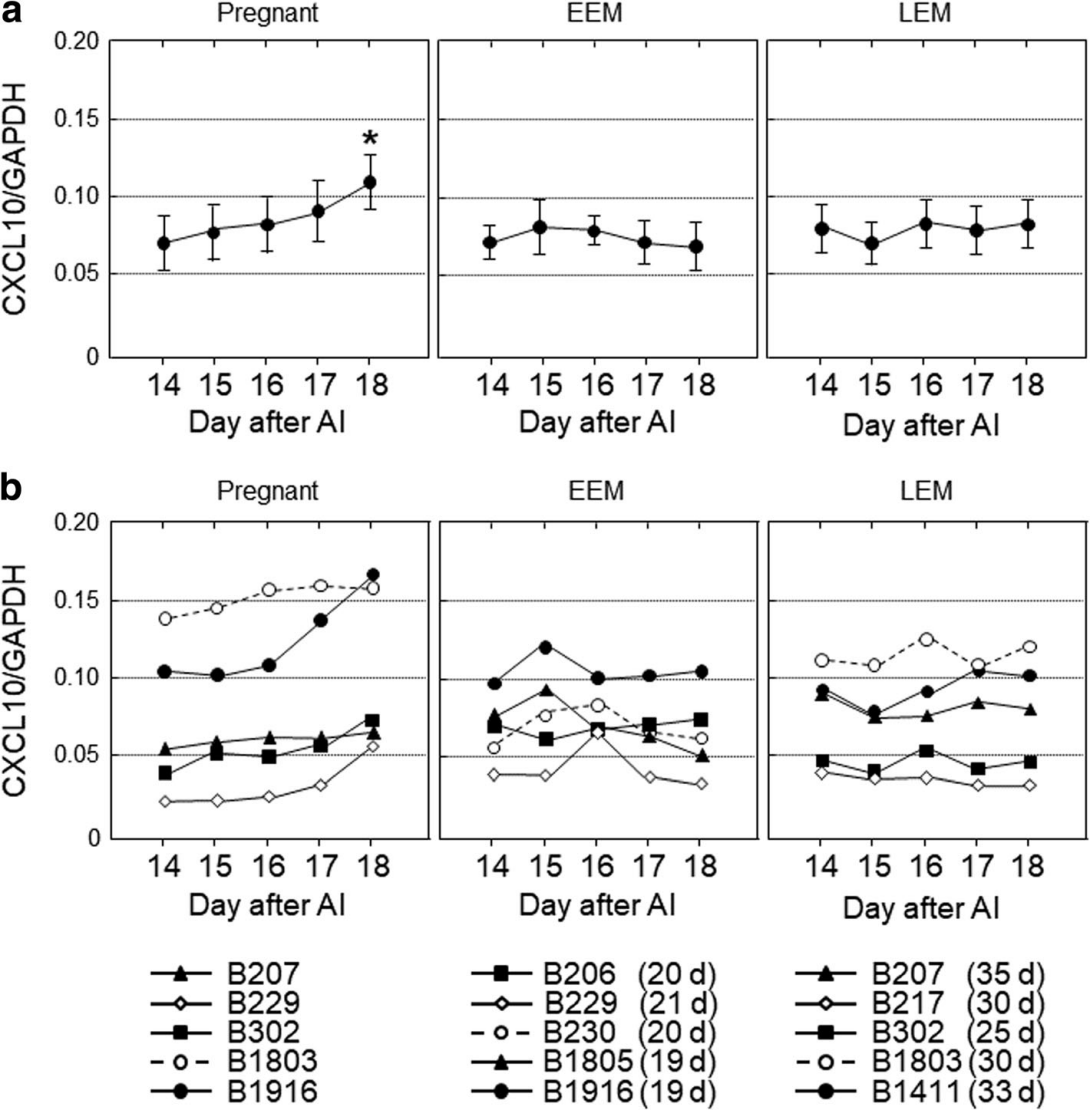
The gene expression levels of *CXCL10* in peripheral blood leukocytes obtained from cows on 14, 15, 16, 17 and 18 d after artificial insemination (AI). Based on the day of return of estrus, the cows were divided into three groups, pregnant (*n* = 5), early embryonic mortality (EEM; *n* = 5) and late embryonic mortality (LEM; *n* = 5). **a** Data are means ± SEM of five cows and are expressed as relative ratios of the mRNAs to *GAPDH*. Values significantly different from the value on 14 d after AI are shown with an asterisk (*P < 0.05). **b** Each line represents the data for individual cows, and the figure in parentheses indicates the day of the return of estrus in EEM and LEM cows

**Fig. 3:**
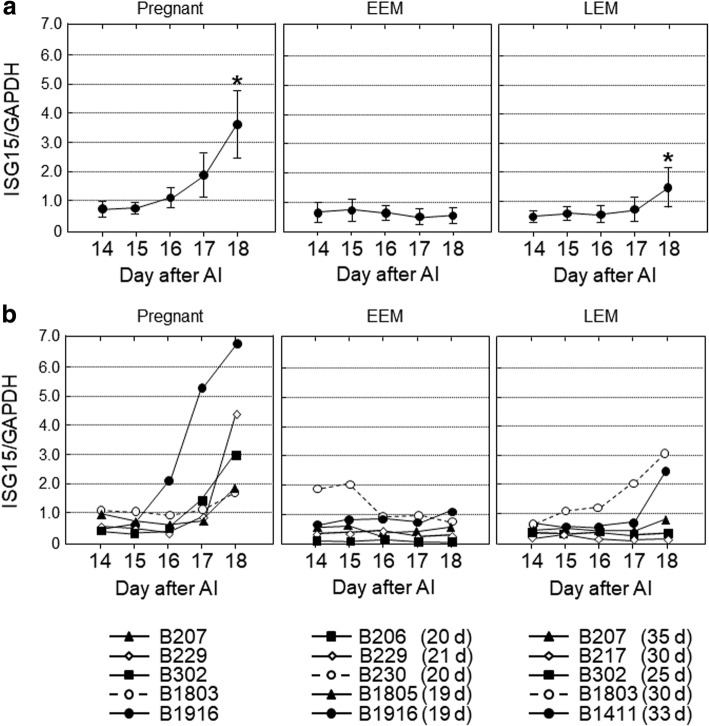
The gene expression levels of *ISG15* in peripheral blood leukocytes obtained from cows on 14, 15, 16, 17 and 18 d after artificial insemination (AI). Based on the day of return of estrus, the cows were divided into three groups, pregnant (*n* = 5), early embryonic mortality (EEM; *n* = 5) and late embryonic mortality (LEM; *n* = 5). **a** Data are means ± SEM of five cows and are expressed as relative ratios of the mRNAs to *GAPDH*. Values significantly different from the value on 14 d after AI are shown with an asterisk (*P < 0.05). **b** Each line represents the data for individual cows, and the figure in parentheses indicates the day of the return of estrus in EEM and LEM cows

**Fig. 4:**
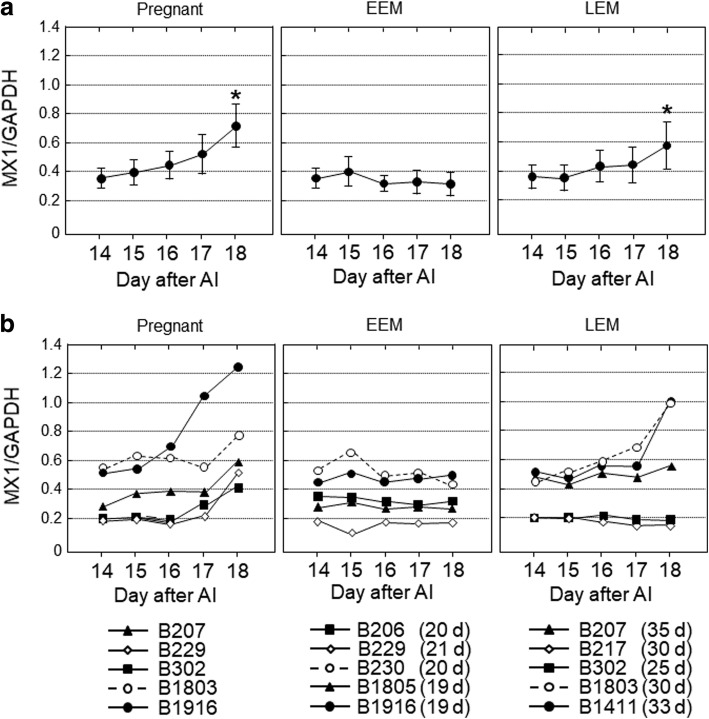
The gene expression levels of *MX1* in peripheral blood leukocytes obtained from cows on 14, 15, 16, 17 and 18 d after artificial insemination (AI). Based on the day of return of estrus, the cows were divided into three groups, pregnant (*n* = 5), early embryonic mortality (EEM; *n* = 5) and late embryonic mortality (LEM; *n* = 5). **a** Data are means ± SEM of five cows and are expressed as relative ratios of the mRNAs to *GAPDH*. Values significantly different from the value on 14 d after AI are shown with an asterisk (*P < 0.05). **b** Each line represents the data for individual cows, and the figure in parentheses indicates the day of the return of estrus in EEM and LEM cows

**Fig. 5:**
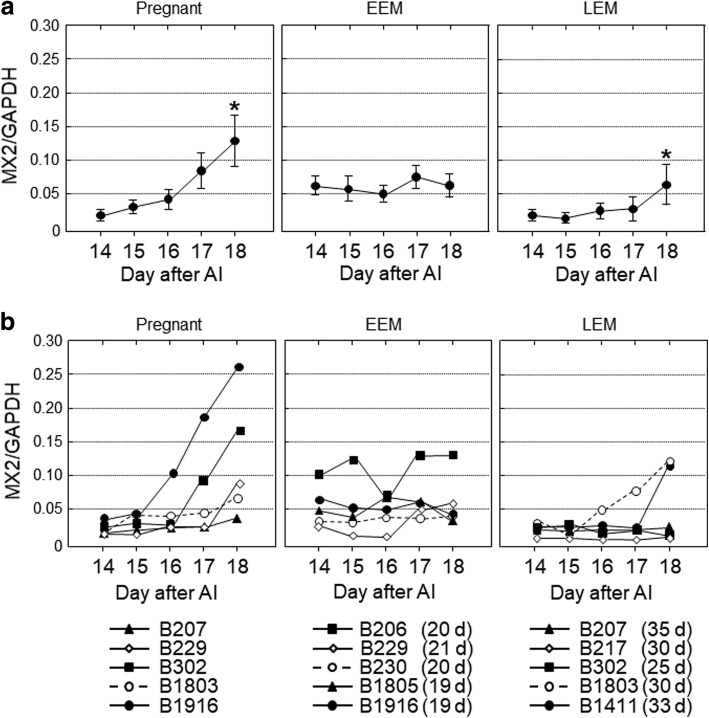
The gene expression levels of *MX2* in peripheral blood leukocytes obtained from cows on 14, 15, 16, 17 and 18 d after artificial insemination (AI). Based on the day of return of estrus, the cows were divided into three groups, pregnant (*n* = 5), early embryonic mortality (EEM; *n* = 5) and late embryonic mortality (LEM; *n* = 5). **a** Data are means ± SEM of five cows and are expressed as relative ratios of the mRNAs to *GAPDH*. Values significantly different from the value on 14 d after AI are shown with an asterisk (*P < 0.05). **b** Each line represents the data for individual cows, and the figure in parentheses indicates the day of the return of estrus in EEM and LEM cows

*CCL8* and *CXCL10* transcripts in PBLs gradually increased from 14 to 18 d of pregnant cows and significant differences were observed on 18 d (Figs. 1 and 2; *P* < 0.05), whereas no significant changes were observed both in EEM and LEM cows. *ISG15*, *MX1* and *MX2* mRNA expression in PBLs was significantly higher on 18 d than 14 d of pregnant cows as well as in LEM cows (Figs. 3, 4, and 5; *P* < 0.05), but no changes were observed in EEM cows.

**Fig. 6:**
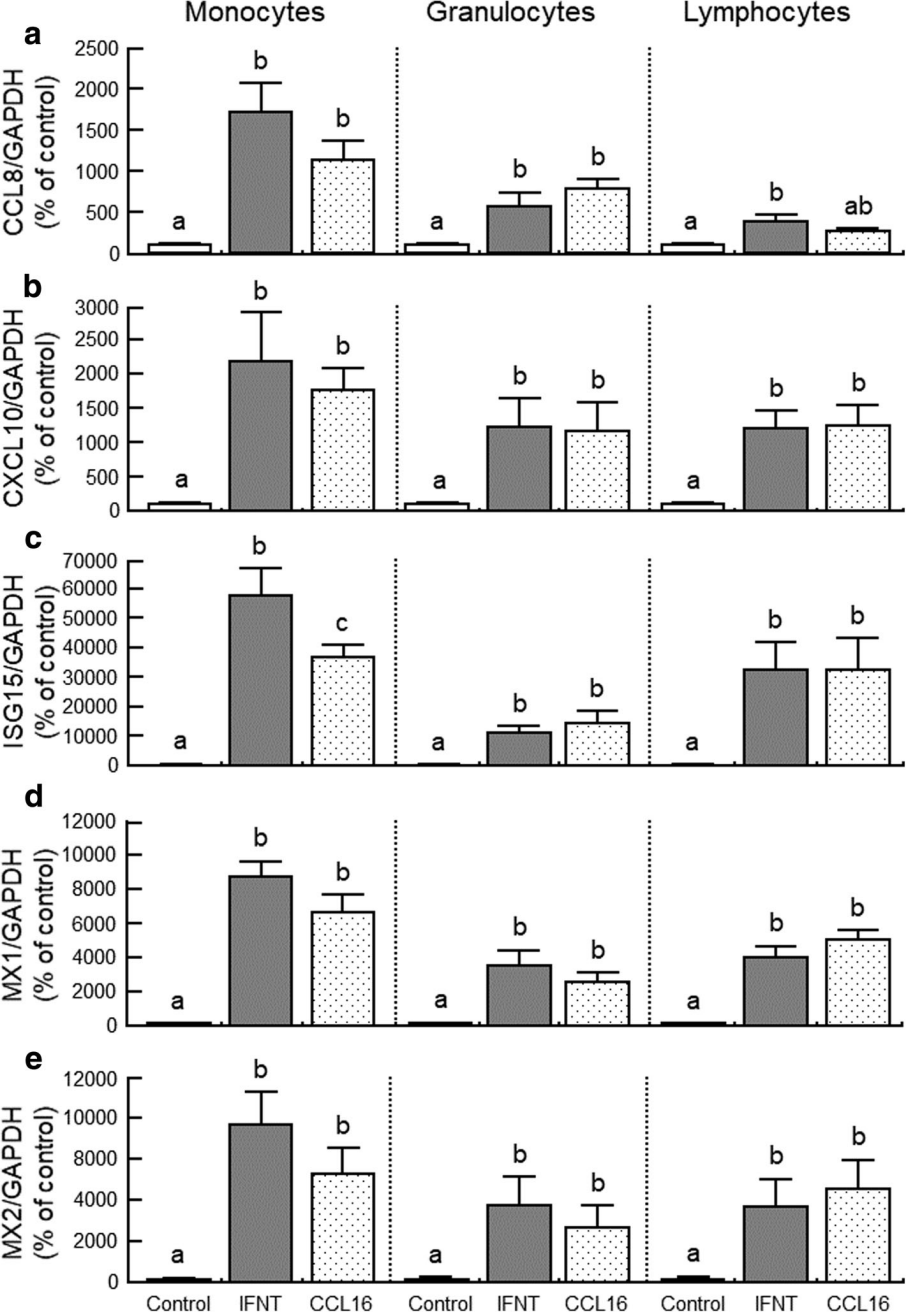
Effects of interferon-τ (IFNT; 100 ng/mL) and CCL16 (100 ng/mL) on the mRNA expression of (**a**) *CCL8*, (**b**) *CXCL10*, (**c**) *ISG15*, (**d**) *MX1* and (**e**) *MX2* in cultured monocytes, granulocytes and lymphocytes. Data are shown as percentage of the control value (*n* = 5). Different letters indicate significant differences (*P* < 0.05)

The addition of IFNT and CCL16 stimulated *CCL8*, *CXCL10*, *ISG15*, *MX1* and *MX2* mRNA expression in cultured monocytes and granulocytes (Fig. 6; *P* < 0.05). The stimulatory effect of IFNT on *ISG15* mRNA expression was higher than CCL16 in monocytes (Fig. 6c; *P* < 0.05). In lymphocytes, IFNT stimulated *CCL8*, *CXCL10*, *ISG15*, *MX1* and *MX2* mRNA expression (Fig. 6; *P* < 0.05). CCL16 stimulated *CXCL10*, *ISG15*, *MX1* and *MX2* mRNA (Fig. 6b-e; *P* < 0.05), but it did not stimulate significantly on *CCL8* mRNA expression (Fig. 6a).

## Discussion

This study demonstrated that the expression of *CCL8* and *CXCL10* genes in PBLs increased on 18 d in pregnant cows, whereas this up-regulation was not observed in non-pregnant cows. In addition, the expression of both *CCL8* and *CXCL10* mRNA in cultured PBLs was stimulated by IFNT. Our previous study shown that the expression of six chemokine genes, including *CCL8* and *CXCL10*, was higher in the bovine endometrium on 15 and 18 d of pregnancy than in the non-pregnant stage, and that *CCL8* and *CXCL10* mRNAs increased after stimulation with IFNT using an in vitro endometrial culture system [15]. These observations support the hypothesis that up-regulation of *CCL8* and *CXCL10* in bovine PBLs is a pregnancy-dependent event.

Chemokines were first discovered as mediators of migration of immune cells to sites of inflammation and injury, and now they play multiple roles in organ development, angiogenesis, and tumorigenesis [19]. Chemokines have also been implicated in a number of reproductive events, such as ovulation, menstruation, embryo implantation, parturition and endometriosis [20, 21]. Peripheral blood cells are mainly composed of erythrocytes, platelets and leukocytes. Leukocytes are further divided into monocytes (macrophages and dendritic cells), granulocytes (neutrophils, eosinophils and basophils) and lymphocytes (T-cells, B-cells and natural killer [NK] cells). CCL8 is produced by monocytes and macrophages, and mainly acts through its receptors (CCR1, CCR2 and CCR3) on monocytes, eosinophils and T lymphocytes [22]. Eosinophils produce CCL8, and the number of eosinophils increases within the uterus during early pregnancy in ewes [23]. The *CCL8* transcript has also been identified in the bovine endometrium and increased in cows during an early stage of pregnancy [15, 24]. Messenger RNA expression of CCL8 receptors are detected in the bovine endometrium during both the estrous cycle and pregnancy, and their proteins are localized in the epithelial and glandular epithelial cells of bovine endometrium [15]. Although CCL8 has been reported to decrease both the expression of cyclooxygenase (COX)-2 and oxytocin receptors in cultured bovine endometrial tissues [15], the physiological importance of CCL8 during maternal recognition still needs to be clarified.

In contrast to CCL8, information on CXCL10 has been well documented [20]. CXCL10 inhibits endothelial cell proliferation, chemotaxis, activation of Th1 cells, NK cells and macrophages. CXCL10 is also known to be induced by interferons and acts in response to several angiogenic factors, including other CXC chemokines and growth factors [25]. CXCL10 is found in monocytes that are localized in the subepithelial stroma of the ovine endometrium, and its expression is stimulated by IFNT and interferon-γ [26]. The level of CXCL10 expression in the endometrium is higher during pregnancy than in the non-pregnant stage in goats [27] and cows [15]. In addition, CXCL10 induces the recruitment of numerous leukocytes in the ovine uterus and enhances the ability of trophoblasts to attach to endometrium [28]. CXCL10 also induces caprine trophoblast adhesion [29] and chemotaxis in human trophoblast cell lines [30]. CXCL10 binds to both CXCR3A and CXCR3B, which are two alternatively spliced forms. CXCR3A is the major chemokine receptor found on T lymphocytes and NK cells, and plays a critical role in the development of anti-tumor immunity and inhibits angiogenesis, which is relevant to a variety of tumors [31], whereas CXCR3B mediates the angiostatic activity of CXCL10 on human microvascular endothelial cells [32]. Thus, IFNT-stimulated CXCL10 production in PBLs may play a variety of roles in many cells including immune cells, endothelial cells, endometrial cells and the conceptus at the time of maternal recognition in cows.

Our previous study demonstrated that *CCL16* mRNA expression was high in the endometrium on 15 d, but not on 18 d, of pregnant cows than in non-pregnant cows. Moreover, since CCL16 stimulated the expression of *ISG15* and *MX1* in cultured endometrial tissues [15], we prospected that CCL16 might affect the expression of chemokines or *ISGs* mRNA in PBLs. In this study, CCL16 stimulated the expression of *CCL8*, *CXCL10* and *ISG* genes in cultured PBLs. CCL16 increases the antigen presentation of macrophages, enhances T-cell cytotoxicity and stimulates the production of a number of inflammatory-type cytokines (IL-1β, TNF, IL-12) [33]. CCL16 and its receptors (CCR1 and CCR2) were identified in the preterm placenta [34] and bovine endometrium [15]. CCL16 induces endothelial cell motility, which is pivotal in vessel formation by stimulating the release of proinflammatory and proangiogenic chemokines [35, 36]. Although a further study is needed to clarify the role of CCL16 in cows, CCL16 may influence angiogenesis and anti-viral activity by up-regulating CCL8 and CXCL10 as well as the expression of ISGs at the time of maternal recognition.

The early diagnosis of pregnancy is useful by shortening interbreeding intervals, and subsequently brings economic benefits for livestock production. Transrectal ultrasonography is commonly used to diagnose pregnancy by visualizing a viable embryo between 28 and 35 d post-AI [1]. Although this method is useful because of its simplicity, earlier diagnostic methods that can be adopted within three or 4 wk are desired. In this study, cows in which estrus returned between 19 and 21 d were classified as EEM, and those between 30 and 35 d as LEM, according to a previous study in which cows were classified as EEM when estrus returned before 24 d, and as LEM when it returned between 24 and 50 d [16]. Since we could not confirm a presence or absence of conceptus by an ultrasound imaging until 30 d after AI, EEM and LEM may not represent early embryonic loss or fertilization failure in fact. The expression of *CCL8* and *CXCL10* genes was high in PBLs on 18 d of pregnant cows, but not in EEM and LEM cows, suggesting that the gene expression profiles of *CCL8* and *CXCL10* may be helpful to estimate embryonic mortality or fertilization failure in cows.

In this study, the expression level of ISGs (ISG15, MX1 and MX2) in the PBLs was higher on 18 d than 14 d in LEM cows as well as pregnant cows, suggesting that the embryo might still be alive on 18 d in LEM cows. However, the expression of *CCL8* and *CXCL10* mRNA did not change in LEM cows. Chemokine production is regulated by growth factors and cytokines/chemokines [25, 26], and indeed the expression of *CCL8* and *CXCL10* mRNAs in cultured PBLs was stimulated by CCL16. These results allow us to hypothesize that up-regulation of chemokines and ISGs in early pregnant cows may in part be regulated by a different pathway. Although *CCL16* was highly expressed in the endometrium on 15 d of pregnant cows than in non-pregnant cows, its expression was not stimulated by IFNT using an in vitro endometrial culture system [15], suggesting that pregnancy-dependent CCL16 upregulation occurs as an indirect action of IFNT. Further studies are needed to clarify the detailed mechanism of chemokine-IFNT interaction in bovine PBLs.

A previous study demonstrated that the expression of *ISGs* in the granulocyte fraction on 14 d of pregnancy was significantly higher than in the monocyte and lymphocyte fractions [13]. Hence, we believe that granulocytes would have more responsibility with IFNT than monocytes and lymphocytes. Unexpectedly, stimulatory effects of IFNT on *ISG15* expression in the monocytes (57,100%) were tended to be high relative to granulocytes (10,943%) and lymphocytes (32,192%) in our in vitro experiments. We could not determine an appropriate reason, but in vitro culture conditions may be the cause for these different results. Although we selected the dose of IFNT (100 ng/mL) and stimulation period (18 h) according to previous studies [15, 37–39], it has also been demonstrated that *ISG15* mRNA expression was stimulated by IFNT at lower concentrations (0.1 and 1 ng/mL) and a shorter period of stimulation (4 h) [40]. Furthermore, IFNT (0.1 ng/mL) for 4 h significantly induced the expression of *ISG15* and *OAS1* in granulocytes, but not in whole PBLs [11]. Therefore, stimulatory effects of IFNT on the expression of ISGs in cultured PBLs might have been masked after 18 h incubation in our in vitro study. On the other hand, we also attempted to measure CCL8, CXCL10, ISG15, MX1 and MX2 proteins using commercial available ELISA kits, since endocrinological early pregnancy diagnosis seems to be a more convenient method than genetic diagnosis. However, the concentration of CCL8, CXCL10 and ISGs in the plasma and conditioned media of cultured PBLs was not detectable and thus it was not possible to evaluate any changes between pregnant and non-pregnant cows. Hence, a very highly sensitive ELISA kit or concentration of target proteins in serum would be needed to apply endocrinological diagnosis in the future.

## Conclusions

The present study demonstrated that the expression of *CCL8* and *CXCL10* genes in PBLs increased from 14 to 18 d of pregnant cows, whereas no significant changes were observed in EEM and LEM cows. Since IFNT stimulated *CCL8* and *CXCL10* expression in cultured PBLs, the increase of *CCL8* and *CXCL10* might be pregnancy-dependent events. Moreover, the expression of both *CCL8* and *CXCL10* in cultured PBLs was stimulated by CCL16 as well as IFNT, suggesting that chemokines, including CCL8, CXCL10 and CCL16, may play some roles at the time of maternal recognition.

## Abbreviations

AI: Artificial insemination
EEM: Early embryonic mortality
ELISA: Enzyme-linked immune solvent assay
IFNT: Interferon-τ
IL: Interleukin
ISGs: Interferon-stimulated genes
LEM: Late embryonic mortality
MX: Myxovirus-resistance gene
OAS1: 2′-5′-oligoadenylate synthetase 1
PBLs: Peripheral blood leukocytes
TNF: Tumor necrosis factor-α
